# A randomized, controlled, double-blind, crossover trial of triheptanoin in alternating hemiplegia of childhood

**DOI:** 10.1186/s13023-017-0713-2

**Published:** 2017-10-02

**Authors:** Elodie Hainque, Samantha Caillet, Sandrine Leroy, Constance Flamand-Roze, Isaac Adanyeguh, Fanny Charbonnier-Beaupel, Maryvonne Retail, Benjamin Le Toullec, Mariana Atencio, Sophie Rivaud-Péchoux, Vanessa Brochard, Florence Habarou, Chris Ottolenghi, Florence Cormier, Aurélie Méneret, Marta Ruiz, Mohamed Doulazmi, Anne Roubergue, Jean-Christophe Corvol, Marie Vidailhet, Fanny Mochel, Emmanuel Roze

**Affiliations:** 1 Université de la Sorbonne, UPMC Paris 06, UMR S 1127, Inserm U 1127, CNRS UMR 7225, Institut du Cerveau et de la Moëlle, F-75013 Paris, France; 2 Département de Neurologie, Groupe Hospitalier Pitié-Salpêtrière, AP-HP, 75013 Paris, France; 3 INSERM, Centre d’Investigation Clinique Neurosciences, CIC-1422, Hôpital Pitié-Salpêtrière, AP-HP, Paris, France; 4 Service de Diététique, Hôpital Pitié-Salpêtrière, AP-HP, Paris, France; 5 EpiScience, London, UK; 6 Centre Hospitalier Sud-Francilien, Université Paris Sud, Corbeil-Essonnes, Service de Neurologie et Unité Neurovasculaire, Corbeil-Essonnes, France; 7 IFPPC, centre CAMKeys, Paris, France; 8 Pharmacie, Hôpital Pitié-Salpêtrière, AP-HP, Paris, France; 9 Service de Biochimie Métabolomique et protéomique, Hôpital Necker et Université Paris Descartes, AP-HP, Paris, France; 10 Sorbonne Universités, UPMC Paris 06, CNRS UMR8256, Institut de Biologie Paris Seine, Adaptation Biologique et vieillissement, Paris, France; 11 Département de Neurologie, Hôpital Saint-Antoine, AP-HP, Paris, France; 12 Département de Génétique, Hôpital Pitié-Salpêtrière, AP-HP, Paris, France; 13 Groupe de Recherche Clinique Neurométabolique, Université Pierre et Marie Curie, Paris, France

**Keywords:** Alternating hemiplegia of childhood, Triheptanoin, Crossover trial

## Abstract

**Background:**

Based on the hypothesis of a brain energy deficit, we investigated the safety and efficacy of triheptanoin on paroxysmal episodes in patients with alternating hemiplegia of childhood due to *ATP1A3* mutations.

**Methods:**

We conducted a randomized, double-blind, placebo-controlled crossover study of triheptanoin, at a target dose corresponding to 30% of daily calorie intake, in ten patients with alternating hemiplegia of childhood due to *ATP1A3* mutations. Each treatment period consisted of a 12-week fixed-dose phase, separated by a 4-week washout period. The primary outcome was the total number of paroxysmal events. Secondary outcomes included the number of paroxysmal motor-epileptic events; a composite score taking into account the number, severity and duration of paroxysmal events; interictal neurological manifestations; the clinical global impression-improvement scale (CGI-I); and safety parameters. The paired non-parametric Wilcoxon test was used to analyze treatment effects.

**Results:**

In an intention-to-treat analysis, triheptanoin failed to reduce the total number of paroxysmal events (*p* = 0.646), including motor-epileptic events (*p* = 0.585), or the composite score (*p* = 0.059). CGI-I score did not differ between triheptanoin and placebo periods. Triheptanoin was well tolerated.

**Conclusions:**

Triheptanoin does not prevent paroxysmal events in Alternating hemiplegia of childhood. We show the feasibility of a randomized placebo-controlled trial in this setting.

**Trial registration:**

The study has been registered with clinicaltrials.gov (NCT002408354) the 03/24/2015.

## Background

Alternating hemiplegia of childhood (AHC) is a rare, early-onset neurodevelopmental disorder. Typically, AHC patients have a baseline neurological disorder with intellectual disability and permanent motor manifestations, along with various paroxysmal episodes [1–6]. *ATP1A3* is the main culprit gene, accounting for about 80–90% of cases of AHC [1, 5, 6].

The *ATP1A3* gene is largely expressed within the brain [7, 8]. It encodes the α3 catalytic subunit of a transmembrane sodium-potassium pump that plays a critical role in generating and maintaining Na^+^ and K^+^ gradients across the membrane of neurons [9, 10]. Disease-causing mutations result in lower Na^+^/K^+^ ATP_ase_ activity, thereby altering neurotransmitter release, neuronal excitability, and intracellular signaling [9–11].

Paroxysmal episodes are characterized by a variable combination of motor deficits, movement disorders, oculomotor abnormalities, pain, autonomic disorders, and seizures [2–4]. Their frequency, severity and duration are highly variable, both in a given patient and from one patient to another. They are often triggered by intense emotions, sensory stimuli such as temperature and light changes, fasting, and physical problems such as infections and trauma [2–4, 12]. Paroxysmal manifestations markedly undermine these patients’ quality of life, and their treatment is highly problematic [13]. The mechanism linking Na^+^/K^+^ ATP_ase_ to paroxysmal events in AHC is largely unknown. Both clinical and radiological data have raised the possibility that a transient brain energy deficit might play a critical role for the paroxysmal manifestations of AHC. Clinical observation found similar triggering factors in AHC and in obvious disorders of brain energy metabolism such as GLUT1 deficiency syndrome [12, 14, 15]. Imaging studies described cerebral glucose hypometabolism on interictal period in human [16] and in a AHC mouse model (Myshkin mice) [17]. Furthermore, ketogenic diet, which efficiently compensates for defective cerebral glucose metabolism, has shown some efficacy on AHC paroxysmal episodes [14, 15, 18].

Triheptanoin (UX007; Ultragenyx Pharmaceuticals Inc.; Novato; USA) is a medium odd-chain triglyceride containing three 7-carbon fatty acids. Its metabolism yields appropriate substrates for both fatty acid metabolism and anaplerosis. Triheptanoin is well tolerated and has been shown to improve clinical manifestations and/or brain metabolism in various disorders associated with patent brain energy deficits, such as glucose transporter deficiency [19], pyruvate carboxylase deficiency [20], and Huntington’s disease [21].

The aim of this randomized, double-blind, placebo-controlled crossover study was to assess the safety and efficacy of triheptanoin on paroxysmal episodes in patients with alternating hemiplegia of childhood due to *ATP1A3* mutations.

## Methods

### Study design and intervention

**Fig. 1:**
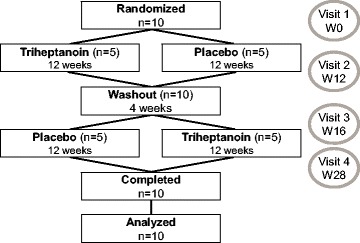
Flow chart of trial participants

This was a randomized, double-blind, placebo-controlled crossover study. All study visits took place at the Clinical Investigation Center for Neurosciences at the Brain and Spine Institute of Pitié-Salpêtrière Hospital between March 2015 and May 2016. The crossover study comprised two treatment periods, one with triheptanoin and the other with placebo (safflower oil with an indistinguishable taste and identical packaging), separated by a 4-week washout period (Fig. 1).

Each treatment period consisted of a 12 (± 2)-week fixed-dose phase, except that only half the daily dose was given on the first day. A trained dietician determined the daily calorie intake for each patient and adjusted his or her daily menu to ensure both proper treatment administration and an isocaloric diet. The patients were asked to ingest a treatment dose (triheptanoin or placebo) representing around 30% of their usual daily calorie intake. The target dose corresponding to 30% of daily calorie intake was defined by metabolism analysis in rodent study [22, 23] and previous clinical trials in human [19, 24]. Treatment was administered during meals, three to four times a day. Treatment was discontinued abruptly for the 4-week washout period, during which the patients resumed their habitual diet. We chose this long washout period to allow rest for the patients between visits 2 and 3. Dietary and therapeutic adherence was assessed by the dietician at visits 2 and 4.

### Outcome measures

Demographic data were collected at the beginning of the study, along with information on the *ATP1A3* mutation. Clinical and biological assessments were done at each visit. Neurological paroxysmal events related to AHC were assessed during each study phase, based on a comprehensive daily diary kept by the patient and/or primary caregiver. All paroxysmal events were recorded, whether motor (palsy, stiffness, oculomotor or limb movement disorders, dysarthria) seizure or non-motor (headache, fatigue, mood swings), along with their severity (scored 0–3) and their approximate duration in minutes, as previously described [19]. If a patient had a combination of two motor manifestations within one event, it was counted as one event. A remission period of 15 min during full wakefulness was arbitrarily used to define two different events. A non-motor manifestation was therefore considered as part of a single event if it started within 15mn after the end of the motor event or if it ended within 15 mn before the motor event. At each visit, the evaluating physician reviewed all paroxysmal events. Clinical changes were assessed with the Clinical Global Impression-Improvement Scale (CGI-I, range 0–7), completed both by the physician and by the patient.

Safety was assessed at each visit by means of an adverse events (AE) questionnaire, as well as bodyweight measurement and laboratory tests (serum electrolytes, blood cell counts, hepatic and renal function tests, plasma C5-keto acids [19]). Hypnosis was offered to support smooth running of blood sampling procedures. Blood was collected in the morning, after an overnight fast (visits 1 and 3), or 90 min after the last intake of the study medication (visits 2 and 4).

The primary endpoint was the total number of paroxysmal events during the triheptanoin and placebo periods. Secondary outcomes were the number of motor-epileptic paroxysmal events, a composite score taking into account both the number of episodes and their severity and duration (cf. equation), interictal neurological manifestations, and the CGI-I.

$$ Composite score=\frac{\sum_{i=0}^n{Severity}^{\ast } Duration of the most severe symptom}{Number of weeks} $$

### Biochemical analyses

Here, “C5 keto acids” refers to the two species of 5-carbon-unit monocarboxylic acids carrying either 3-keto or 3-hydroxy radicals. Their plasma levels were measured by organic extraction, trimethylsilylation (BSTFA +1% TMCS, Sigma) and gas chromatography-mass spectrometry in SIM mode (Scion TQ mass analyzer, Brüker). Quantification was calibrated on known amounts of unlabeled analytes relative to stable-isotope-labeled internal standards (3,4,5-1^3^C_3_ 3-ketopentanoate from Eurisotop, Saint Aubin, France and 2,2,3,3,4,4,5,5,6,6-d_10_ 6-hydroxyhexanoic acid from Sigma-Aldrich). The concentrations of plasma C3-carnitine, produced only by triheptanoin due to its odd number of carbons, were also measured as described [20].

### Study population

We enrolled AHC patients older than 15 years who had a proven *ATP1A3* mutation, had at least six paroxysmal events during the 3 months prior to enrollment, and were on a normal diet.

Patients were excluded if they had past or present severe psychiatric disorders; comorbid medical conditions that would render them unsuitable for the study; cognitive impairment preventing full understanding of the study; and, for women of child-bearing potential, pregnancy, breastfeeding and nonuse of effective dual contraception. Drugs with a possible effect on alternating hemiplegia were allowed, at stable doses, throughout the study.

### Randomization and blinding

A computer-generated randomization plan (www.randoweb@aphp.fr) was used for patient assignment to one of the two treatment sequences, namely triheptanoin followed by placebo, and placebo followed by triheptanoin. For each patient, an inclusion number was provided to the investigators upon connection to the randomization platform. Each inclusion number corresponded to an individual sequence and the patient remained in it throughout the study. Only the pharmacists were aware of the sequence, and dispensed triheptanoin or placebo accordingly. The pharmacists were also responsible for the unblinding process if required by the safety committee. All patients, caregivers and investigators were blinded to the treatment allocation. Triheptanoin and placebo were supplied by Ultragenyx Pharmaceuticals Inc.; they had an identical aspect and were administered in identical amounts.

### Statistical analysis

Owing to a lack of previous clinical trials, no efficacy data were available. For the sample size calculation, we postulated a reduction in the total number of paroxysmal events of at least 40% with triheptanoin versus placebo. A sample of 10 patients was required to detect this difference with 80% power at a significance level of 5%.

Efficacy was analyzed on an intention-to-treat (ITT) basis. The ITT population included all randomized patients. The treatment effect was evaluated by comparing the treatment and placebo periods using the paired nonparametric Wilcoxon test, taking into account the small sample size and assuming a non Gaussian distribution. Paired nonparametric Wilcoxon tests were used to verify the absence of a carryover effect. The duration of paroxysmal events was not reported in 6% of cases, and these events were omitted from the calculation of the composite score.

### Role of the funding source

This study was supported by the French Association of Alternating Hemiplegia of Childhood (AFHA) and by the fund from AFER for medical research. Ultragenyx provided the triheptanoin and placebo oils. Ultragenyx was not involved in the study design, conduct, monitoring, data analysis or manuscript preparation.

## Results

### Participants

Ten patients were enrolled in the study (Table 1, Fig. 1). All the patients had a proven mutation in the *ATP1A3* gene. Eight patients received a fixed dose of flunarizine (mean 9.4 mg, standard deviation [SD] 4.9 mg). Mean daily calorie intake was 2166 kcal (SD 354 kcal). Lipids comprising triheptanoin/placebo oil and a small portion of lipids intake from other sources (daily diet) represented 38% of daily calorie intake. On average, triheptanoin/placebo oil represented 26% of daily calorie intake (target 30%). All the patients were considered compliant with the study treatments, consuming 87% of the recommended triheptanoin/placebo dose (treatment compliance (%) = consumed dose × 100 / prescribed dose).

**Table 1:** Baseline characteristics of the trial participants

Variable	Total (*n* = 10)
Age at inclusion, y, median (IQR)	18.7 (17.9–20.2)
Male sex, n (%)	4 (40)
Age at diagnosis, m, median (IQR)	6.9 (0.1–11.4)
Mutation of *ATP1A3*	
*p.D801N*	5 (50)
Other mutations	5 (50)
Total paroxysmal events per week	
Median (IQR)	3.88 (3.19–4.85)
Mean (SD)	4.39 (1.95)
Chronic medications, n (%)	9 (90)
Number, median (IQR)	3 (3–4)
Flunarizine, n (%)	8 (80)
Acetazolamide, n (%)	2 (20)
Permanent neurologic deficiency	
Pyramidal syndrome	8 (80)
Cerebellar syndrome	8 (80)
Dystonia	8 (80)
Cognitive/ behavioral dysfunction	6 (60)

n (%) represents the number of patients (and the percentage of all patients) assessed in each group. *Abbreviations*: *Y* years, *m* months, *IQR* interquartile range [p25–p75]

### Treatment effects

Changes in the primary and secondary outcome variables are shown in Table 2.

**Table 2:** Changes in primary and secondary outcome variables

Variable	Triheptanoin	Placebo	*p* value
Total paroxysmal events	3.5 (2.1)	3.2 (2.1)	0.646
Motor-epilepticparoxysmal events	3.4 (1.9)	3.2 (2.0)	0.585
Composite score	1015 (1058)	723.9 (767.3)	0.059
CGI – patient	3.7 (1.5)	3.2 (1.0)	0.481
CGI – physician	3.8 (0.6)	3.3 (0.9)	0.262

Values are means (standard deviation). Total and motor-epileptic paroxysmal events are expressed per week. Wilcoxon test for the difference in changes on each treatment. *Abbreviations*: *CGI* Clinical Global Impression-Improvement scale

Triheptanoin did not reduce the total number of paroxysmal events. Triheptanoin also failed to reduce the number of motor-epileptic paroxysmal events, and the composite score. The CGI-I patient and physician scores were both unchanged between the triheptanoin and placebo periods. There was no difference in interictal neurological manifestations. No carryover effect was detected.

### Biochemical analyses

Plasma C3-carnitine measured at the end of each period were significantly higher with triheptanoin than with placebo (1.56 μmol/L versus 0.32 μmol/L; *p* < 0.01). C5-keto acids levels measured at the end of each period were significantly higher with triheptanoin than with placebo (3-hydroxypentanoate 34.6 μmol/L, 3-ketopentanoate 12.2 μmol/L, versus 0.2 and 0.2 μmol/L, respectively; *p* < 0.01). These results reflected the proper metabolism of triheptanoin.

### Safety

Nineteen adverse events (AE) were reported, of which 5 occurred during the washout period. Fifty percent of patients reported an AE on triheptanoin and 70% on placebo. The AEs corresponding to each treatment period are listed in Table 3. All AEs were transient. Two serious AEs were reported (generalized seizures requiring hospitalization), one during triheptanoin administration and one during the washout period. No study withdrawals occurred. Weight and the body mass index remained stable during the study. Routine laboratory tests showed no noteworthy changes during the study (data not shown).

**Table 3:** Adverse events

Adverse event	Triheptanoin (*n* = 6)	Placebo (*n* = 8)	WO (*n* = 5)
Infection	3 (50)	5 (63)	3 (60)
Digestive disorders	1 (17)	2 (25)	0 (0)
Others	2 (33)	1 (13)	2 (40)

Data are n (%). The denominator is the number of trial participants who had adverse events. *Abbreviation*: *AE* adverse events, *WO* washout period

## Discussion

This randomized, double-blind, placebo-controlled crossover trial failed to show any effect of triheptanoin, at a target dose of 30% of calories, on paroxysmal episodes in patients with alternating hemiplegia of childhood. This argues against a prominent role of a brain energy deficit in these paroxysmal manifestations. Many lessons for future trials in this group of patients were learned during this study.

Many lessons for future trials in this group of patients were learned during this study. These treatments were evaluated in open-label studies or case series. Prophylactic treatment of paroxysmal episodes is a major issue in alternating hemiplegia of childhood. Flunarizine is considered partly effective in some patients but rarely abrogates the attacks. Some degree of improvement is reported in 60–80% of patients in large retrospective series [2, 4, 16, 18, 22, 25]. A broad range of medications (antimigraine drugs and antiepileptics, particularly benzodiazepines) have been tried, mostly prophylactically, with limited efficacy and/or poor tolerability [2, 4, 13]. These treatments were evaluated in open-label studies or case series. However, the only available controlled study testing flunarizine in nine AHC patients was not conclusive, likely due to methodological issues [26]. Our study demonstrates the feasibility of a controlled trial in AHC. Treatment with triheptanoin was not effective to prevent paroxysmal episodes in AHC. Adverse effect were similar in the treatment and placebo groups further indicating that triheptanoin is well tolerated, even in patients with severe neurological disorders.

Our study was based on the hypothesis that paroxysmal episodes are linked to a defective brain energy supply. The following observations are consistent with this hypothesis: i) paroxysmal episodes in AHC share some common triggering factors seen in patent disorders of brain energy metabolism such as glut1 deficiency syndrome [2, 12, 23, 24, 27]; ii) attacks can generally be terminated by inducing sleep that might reflect a reduced brain energy demand [2, 12]; iii) an anecdotal report mentions the sustained disappearance of paroxysmal episodes on a ketogenic diet, which efficiently compensates for defective brain glucose metabolism [14, 15, 18]; iv) neuroimaging studies of interictal brain glucose metabolism showed focal areas of reduced glucose metabolism in AHC patients [16] and in an AHC mouse model [17]; and v) Na^+^/K^+^ ATPase dysfunction might influence sodium-dependent brain glucose transportation, particularly in situations where brain energy demand is increased [28–30]. Triheptanoin has been shown to improve cerebral bioenergetics in various conditions associated with brain energy defects [19, 20, 31, 32]. Its failure to improve paroxysmal episodes in our study does not support a role for cerebral energetic dysfunction in the pathogenesis of AHC. The prevailing hypothesis rather remains that the pump cannot normally utilize ATP or maintain a normal Na+ /K+ gradient.

Although randomized controlled trials are challenging in AHC, they are needed to avoid unnecessary exposure of patients to multiple empirical drug trials. Knowledge and experience gained during the planning, execution and analysis of this study may prove valuable for future controlled studies in this setting: relevant issues include funding, patient recruitment, trial design and duration, and outcome measures. The main financial support provided by the French patients’ association for alternating hemiplegia of childhood (AFHA) was critical, as industrial and institutional funding is rarely forthcoming in very rare diseases. AFHA also enabled us to recruit patients from a large geographic area. To optimize recruitment while avoiding exposure of young children to the constraints of a therapeutic trial, we set the minimal age at 15 years. The phenomenology of paroxysmal episodes is different in paediatric and adult patients [2]. Therefore, our results may not be fully relevant for the paedriatic AHC population. The crossover design also reduced the required number of patients. Given the rarity of AHC, a parallel study design would have increased the risk of baseline differences between the treated and control groups, despite randomization. To limit possible bias due to spontaneous fluctuations in disease severity, we chose relatively long treatment periods (12 weeks) to quantify paroxysmal episodes. To avoid drop-outs, we paid special attention to transport and accommodation during study visits. As AHC patients are extremely vulnerable to stress and change, we allocated extra time for examinations, investigations and discussions, and we proposed hypnotherapy when potentially beneficial. The primary outcome measure was the number of paroxysmal events, as assessed by the patient (or caregiver) and recorded in a handwritten diary. A collection bias is likely thus have occurred although this bias may have been limited by the use of a crossover design (patient is his/her own control). Even for trained experts, it is sometimes challenging to properly characterize the paroxysmal events in AHC. In particular, standardized classification of paroxysmal events with intermingled manifestations is problematic and it may be difficult to distinguish between epileptic seizures and non-epileptic motor events. We therefore choose to study the total number of paroxysmal events as a primary outcome. The main drawback of this approach is a risk of overlooking an effect of the intervention on a specific type of paroxysmal event. Moreover, there are no dedicated tools for evaluating paroxysmal events in this setting. Yet, some data were missing in our study for 6% of episodes. The use of an electronic diary with automated reminders might favor more comprehensive data collection. The functional consequences of each paroxysmal event might be more relevant, along with a separate evaluation of associated pain. Our methodology is a start point and needs to be built on given the above limitations.

## Conclusion

Triheptanoin does not prevent paroxysmal events in alternating hemiplegia of childhood. Our findings do not support a prominent role of a brain energy deficit in alternating hemiplegia. However, randomized placebo-controlled trials are feasible in alternating hemiplegia.

## Abbreviations

AE: Adverse events
AFHA: French Association of Alternating Hemiplegia of Childhood
AHC: Alternating hemiplegia of childhood
CGI-I: Clinical Global Impression-Improvement Scale
ITT: Intention-to-treat
SD: Standard deviation

## References

[1] Heinzen EL, Swoboda KJ, Hitomi Y, Gurrieri F, Nicole S, de Vries B (2012). De novo mutations in ATP1A3 cause alternating hemiplegia of childhood. Nat Genet.

[2] Panagiotakaki E, Gobbi G, Neville B, Ebinger F, Campistol J, Nevsímalová S (2010). Evidence of a non-progressive course of alternating hemiplegia of childhood: study of a large cohort of children and adults. Brain.

[3] Panagiotakaki E, De Grandis E, Stagnaro M, Heinzen EL, Fons C, Sisodiya S (2015). Clinical profile of patients with ATP1A3 mutations in Alternating Hemiplegia of Childhood-a study of 155 patients. Orphanet J Rare Dis.

[4] Sweney MT, Silver K, Gerard-Blanluet M, Pedespan J-M, Renault F, Arzimanoglou A (2009). Alternating hemiplegia of childhood: early characteristics and evolution of a neurodevelopmental syndrome. Pediatrics.

[5] Rosewich H, Thiele H, Ohlenbusch A, Maschke U, Altmüller J, Frommolt P (2012). Heterozygous de-novo mutations in ATP1A3 in patients with alternating hemiplegia of childhood: a whole-exome sequencing gene-identification study. Lancet Neurol.

[6] Ishii A, Saito Y, Mitsui J, Ishiura H, Yoshimura J, Arai H (2013). Identification of ATP1A3 mutations by exome sequencing as the cause of alternating hemiplegia of childhood in Japanese patients. PLoS One.

[7] McGrail KM, Phillips JM, Sweadner KJ (1991). Immunofluorescent localization of three Na,K-ATPase isozymes in the rat central nervous system: both neurons and glia can express more than one Na,K-ATPase. J Neurosci.

[8] Bottger P, Tracz Z, Heuck A, Nissen P, Romero-Ramos M, Lykke-Hartmann K (2011). Distribution of Na/K-ATPase alpha 3 isoform, a sodium-potassium P-type pump associated with rapid-onset of dystonia parkinsonism (RDP) in the adult mouse brain. J Comp Neurol.

[9] Holm R, Toustrup-Jensen MS, Einholm AP, Schack VR, Andersen JP, Vilsen B (2016). Neurological disease mutations of α3 Na(+),K(+)-ATPase: Structural and functional perspectives and rescue of compromised function. Biochim Biophys Acta.

[10] Kinoshita PF, Leite JA, Orellana AMM, Vasconcelos AR, Quintas LEM, Kawamoto EM (2016). The Influence of Na(+), K(+)-ATPase on Glutamate Signaling in Neurodegenerative Diseases and Senescence. Front Physiol.

[11] Heinzen EL, Arzimanoglou A, Brashear A, Clapcote SJ, Gurrieri F, Goldstein DB (2014). Distinct neurological disorders with ATP1A3 mutations. Lancet Neurol.

[12] Rosewich H, Baethmann M, Ohlenbusch A, Gärtner J, Brockmann K (2014). A novel ATP1A3 mutation with unique clinical presentation. J Neurol Sci.

[13] Neville BGR, Ninan M (2007). The treatment and management of alternating hemiplegia of childhood. Dev Med Child Neurol.

[14] Vila-Pueyo M, Pons R, Raspall-Chaure M, Marcé-Grau A, Carreño O, Sintas C (2014). Clinical and genetic analysis in alternating hemiplegia of childhood: ten new patients from Southern Europe. J Neurol Sci.

[15] Roubergue A, Philibert B, Gautier A, Kuster A, Markowicz K, Billette de Villemeur T (2015). Excellent response to a ketogenic diet in a patient with alternating hemiplegia of childhood. JIMD Rep.

[16] Sasaki M, Sakuma H, Fukushima A, Yamada K, Ohnishi T, Matsuda H (2009). Abnormal cerebral glucose metabolism in alternating hemiplegia of childhood. Brain and Development.

[17] Kirshenbaum GS, Dawson N, Mullins JGL, Johnston TH, Drinkhill MJ, Edwards IJ (2013). Alternating hemiplegia of childhood-related neural and behavioural phenotypes in Na+,K+−ATPase α3 missense mutant mice. PLoS One.

[18] Pisciotta L, Gherzi M, Stagnaro M, Calevo MG, Giannotta M, Vavassori MR, et al. Alternating Hemiplegia of Childhood: Pharmacological treatment of 30 Italian patients. Brain Dev [Internet] [cited 8 Mar 2017]; Available from: http://www.sciencedirect.com/science/article/pii/S0387760417300281 10.1016/j.braindev.2017.02.001 28249736

[19] Mochel F, Hainque E, Gras D, Adanyeguh IM, Caillet S, Héron B (2016). Triheptanoin dramatically reduces paroxysmal motor disorder in patients with GLUT1 deficiency. J Neurol Neurosurg Psychiatry.

[20] Mochel F, DeLonlay P, Touati G, Brunengraber H, Kinman RP, Rabier D (2005). Pyruvate carboxylase deficiency: clinical and biochemical response to anaplerotic diet therapy. Mol Genet Metab.

[21] Adanyeguh IM, Rinaldi D, Henry P-G, Caillet S, Valabregue R, Durr A (2015). Triheptanoin improves brain energy metabolism in patients with Huntington disease. Neurology.

[22] Gu L, Zhang G-F, Kombu RS, Allen F, Kutz G, Brewer W-U (2010). Parenteral and enteral metabolism of anaplerotic triheptanoin in normal rats. II. Effects on lipolysis, glucose production, and liver acyl-CoA profile. Am J Physiol Endocrinol Metab.

[23] Kinman RP, Kasumov T, Jobbins KA, Thomas KR, Adams JE, Brunengraber LN (2006). Parenteral and enteral metabolism of anaplerotic triheptanoin in normal rats. Am J Physiol Endocrinol Metab.

[24] Roe CR, Sweetman L, Roe DS, David F, Brunengraber H (2002). Treatment of cardiomyopathy and rhabdomyolysis in long-chain fat oxidation disorders using an anaplerotic odd-chain triglyceride. J Clin Invest.

[25] Mikati MA, Kramer U, Zupanc ML, Shanahan RJ (2000). Alternating hemiplegia of childhood: clinical manifestations and long-term outcome. Pediatr Neurol.

[26] Casaer P (1987). Flunarizine in alternating hemiplegia in childhood. An international study in 12 children. Neuropediatrics.

[27] Méneret A, Roze E (2016). Paroxysmal movement disorders: An update. Rev Neurol.

[28] Skou JC (1998). Nobel Lecture. The identification of the sodium pump. Biosci Rep.

[29] Yu AS, Hirayama BA, Timbol G, Liu J, Diez-Sampedro A, Kepe V (2013). Regional distribution of SGLT activity in rat brain in vivo. Am J Physiol Cell Physiol.

[30] Clapcote SJ, Duffy S, Xie G, Kirshenbaum G, Bechard AR, Rodacker Schack V (2009). Mutation I810N in the alpha3 isoform of Na+,K+−ATPase causes impairments in the sodium pump and hyperexcitability in the CNS. Proc Natl Acad Sci U S A.

[31] Gras D, Roze E, Caillet S, Méneret A, Doummar D (2014). Billette de Villemeur T, et al. GLUT1 deficiency syndrome: an update. Rev Neurol.

[32] Roe CR, Bottiglieri T, Wallace M, Arning E, Martin A (2010). Adult Polyglucosan Body Disease (APBD): Anaplerotic diet therapy (Triheptanoin) and demonstration of defective methylation pathways. Mol Genet Metab.

